# Full Breastfeeding and Allergic Diseases—Long-Term Protection or Rebound Effects?

**DOI:** 10.3390/nu15122780

**Published:** 2023-06-16

**Authors:** Lars Libuda, Birgit Filipiak-Pittroff, Marie Standl, Tamara Schikowski, Andrea von Berg, Sibylle Koletzko, Carl-Peter Bauer, Joachim Heinrich, Dietrich Berdel, Monika Gappa

**Affiliations:** 1 Institute of Nutrition, Consumption and Health, Faculty of Natural Sciences, Paderborn University, Warburger Straße 100, 33098 Paderborn, Germany; 2 Children‘s Hospital, Evangelisches Krankenhaus Düsseldorf, 40217 Düsseldorf, Germany; 3 Formerly Department of Pediatrics, Research Institute, Marien-Hospital Wesel, 46483 Wesel, Germany; 4 Institute of Epidemiology, Helmholtz Zentrum München, German Research Center for Environmental Health, 85764 Neuherberg, Germany; 5 Comprehensive Pneumology Center Munich (CPC-M), German Center for Lung Research (DZL), 81377 Munich, Germany; 6 IUF-Leibniz Research Institute for Environmental Medicine, 40225 Düsseldorf, Germany; 7 Department of Pediatrics, Dr. von Hauner Children’s Hospital, University Hospital, LMU Munich, 80337 Munich, Germany; 8 Department of Pediatrics, Gastroenterology and Nutrition, School of Medicine Collegium Medicum, University of Warmia and Mazury, 10-719 Olsztyn, Poland; 9 Department of Pediatrics, Technical University of Munich, 80804 Munich, Germany; 10 Institute and Clinic for Occupational, Social and Environmental Medicine, University Hospital, LMU Munich, 80336 Munich, Germany; 11 Allergy and Lung Health Unit, Melbourne School of Population and Global Health, The University of Melbourne, Melbourne 3010, Australia

**Keywords:** GINIplus, breastfeeding, atopic diseases, allergy prevention, early nutrition, long-term effects, rebound

## Abstract

A previous follow-up of the GINIplus study showed that breastfeeding could protect against early eczema. However, effects diminished in adolescence, possibly indicating a “rebound effect” in breastfed children after initial protection. We evaluated the role of early eczema until three years of age on allergies until young adulthood and assessed whether early eczema modifies the association between breastfeeding and allergies. Data from GINIplus until 20-years of age (*N* = 4058) were considered. Information on atopic eczema, asthma, and rhinitis was based on reported physician’s diagnoses. Adjusted Odds Ratios (aOR) were modelled by using generalized estimating equations. Early eczema was associated with eczema (aORs = 3.2–14.4), asthma (aORs = 2.2–2.7), and rhinitis (aORs = 1.2–2.7) until young adulthood. For eczema, this association decreased with age (*p*-for-interaction = 0.002–0.006). Longitudinal models did not show associations between breastfeeding and the respective allergies from 5 to 20 years of age. Moreover, early eczema generally did not modify the association between milk feeding and allergies except for rhinitis in participants without family history of atopy. Early eczema strongly predicts allergies until young adulthood. While preventive effects of full breastfeeding on eczema in infants with family history of atopy does not persist until young adulthood, the hypothesis of a rebound effect after initial protection cannot be confirmed.

## 1. Introduction

Dietary guidelines in Germany recommend exclusive breastfeeding in the first six months of life and an introduction in complementary foods not before the fifth month of life [1]. German guidelines for allergy prevention recently also suggested exclusive breastfeeding for the first four to six month after birth, but this recommendation was not based on evidence of a preventive effect of breastfeeding on allergies, but general health-promoting effects of breastfeeding for mother and child [2]. The evidence regarding allergy prevention was classified as inconsistent considering that the majority of recent studies did not point to protective effects [3,4,5,6,7].

However, the guidelines mention an analysis of the GINIplus study which indicates that the benefits of breastfeeding for allergy prevention might be restricted to children with a family history of atopy in first-degree relative, i.e., those children considered at risk to develop an allergic disease [8]. The cumulative incidence of early eczema up to three years of age was lower in high-risk children who were fully breastfed for at least four months compared to their counterparts who received conventional cow’s milk formula (CMF) in this period, either as part of partial breastfeeding or as the only source of food. Protective effects of breastfeeding compared with CMF on atopic eczema during the first years of life were already reported in previous analyses of the GINIplus study [9,10]. However, after age three until 15 years of age, the observed differences in cumulative incidences of eczema in the GINIplus study attenuated [8]. This finding is in line with results from a meta-analysis considering data from 24 cohort studies, 17 cross-sectional studies, and one case-control study [11]. The authors reported protective effects of breastfeeding on eczema until the age of two years of life, but disappearing effects thereafter. Moreover, results from studies covering the age between three and 20 years seem to indicate a slightly increased risk for eczema, although the pooled estimate was not significant. Accordingly, the decrease in group differences in GINIplus upon inclusion of data until 15 years of age might indicate a rebound effect after initial protection, i.e., that eczema manifests more frequently after the age of three years in fully breastfed children without early eczema compared to their counterparts fed with CMF. Following this hypothesis, early eczema would modify the long-term preventive effect of full breastfeeding. Accordingly, breastfed children without early eczema might represent a vulnerable group for allergic disease manifestation with increasing age and would, thus, be a target group for additional preventive measures and regular clinical assessments.

An analysis of the GINI intervention cohort after 20-years follow-up recently demonstrated heterogeneous long-term effects of different infant CMF on allergies [12], but long-term effects of full breastfeeding remain to be evaluated. Moreover, early eczema in the first three years was only considered in pathway analysis for different types of hydrolyzed formulae [12]. Thus, the meaning of early eczema protection for long-term effects of full breastfeeding is still unclear. Extending the analysis to data from the non-intervention cohort in the GINIplus study, we aimed to examine the following questions:

- Is early eczema a determinant of the course of allergic diseases until early adulthood which should be considered in long-term analysis of breastfeeding effects?
- Does early eczema modify potential long-term associations of milk feeding with the development of atopic diseases?

## 2. Materials and Methods

### 2.1. Study Design of the GINIplus Study

The current data analysis considered data from the GINIplus study from birth until the age of 20 years. Details of the study design of the GINIplus study have previously been described [8,13,14,15]. In short, 5991 healthy term newborns were initially recruited from 16 maternity wards in two regions of Germany (rural Wesel and urban Munich) between September 1995 and June 1998 and either participated in the GINI intervention study (I cohort) or the GINI non-intervention study (NI cohort). Mothers with a chronic, immunological relevant disease other than allergies (e.g., HIV, autoimmune disease, diabetes) were excluded [15]. The baseline characteristics from the families (e.g., allergies in parents and siblings and parental education) were assessed by questionnaires at birth or before. Written informed consent was obtained from the participating families. The study protocol was approved by the local ethics committees.

In the prospective, double-blind intervention trial only infants with a high family risk of atopy defined as having a family history (FH+) with at least one parent or biologic sibling with a history of allergic disease were included. If parents agreed to participate in the intervention study (I cohort, *N* = 2252), the newborns were randomly allocated at birth to one of four blinded study formulae, i.e., three different hydrolyzed formulae (partially hydrolyzed whey (pHF-W); extensively hydrolyzed whey (eHF-W); extensively hydrolyzed casein (eHF-C)) and one formula based on intact cow’s milk protein (CMF). Mothers in the I cohort received written recommendations for feeding of the infants. Mothers were, e.g., encouraged to exclusively breastfeed for at least four months and not to introduce solid foods during this period. The respective formula was used during the first four months of life as a milk substitute only if exclusive breastfeeding was not possible [16]. Infants with a negative family history of allergy in a first degree relative (FH−, *N* = 2507) or those with positive risk whose parents did not want to participate in the intervention trial (FH+, *N* = 1232) were allocated to the NI-cohort. Their parents did not receive any feeding recommendations.

### 2.2. Definition of Outcome and Exposure Variables

Both cohorts have regularly been followed from birth onwards and recently participated in the 20-years follow-up. Self-administered questionnaires were sent to the parents around their child’s 1st, 2nd, 3rd, 4th, 6th, 10th, and 15th birthdays and to the study participants themselves around their 20th birthdays to collect information on the child’s health, allergic symptoms, physician diagnoses of allergic diseases, and further information such as nutrition and several lifestyle factors. The definition of the main outcomes in the GINI study was continuously based on the same set of questions, which have been used since the first year of life. Until age 15 years, the following question was asked to the parents separately for each year of life: “Did a doctor diagnose your child with one of the following diseases [eczema, asthma, allergic rhinitis, hay fever] in 1st [2nd, …, 15th] year of life?” For the 20-year-questionnaire covering the period from 16–20 years of age, this question was directly addressed to the participants themselves. We further considered a question on disease treatment (“Have you (your child) been treated for [asthma, hay fever, allergic rhinitis, atopic eczema] in the past 12 months?”).

The primary outcomes of the present analyses are period prevalence of eczema, asthma, and allergic rhinitis/hay fever as well as the cumulative incidence of these up to 20 years of age. Any “yes” response to physician’s diagnosis in the period and/or treatment in the last 12 months was used to determine period prevalence. Any positive reply during the lifetime of the child was used to determine cumulative incidence. Early eczema was defined considering physician’s diagnosis of eczema in the 1st, 2nd, and 3rd year of life as answered by the parents in the respective questionnaires.

In the I-cohort, information on milk feeding was derived from weekly diaries. Full breastfeeding was defined if “breast milk only” was reported as milk feeding for each of the first 16 weeks. Accordingly, one documented bottle of the randomized study formula sufficed for the definition “mixed fed with breast milk and study formula”. In the NI-cohort the mode of milk feeding was retrospectively assessed at the age of 1 year using the question “What kind of milk did your child drink during 1st, 2nd, … 6th month of life?” The child was classified as “fully breastfed” if parents selected “breast milk only” for all of months 1 to 4, infants receiving formula during this period were labelled as “not fully breastfed”.

### 2.3. Statistics

Statistical analyses were performed using the statistical software SAS for Windows, Release 9.4 (SAS Institute, Cary, NC, USA). To determine associations between the status of early eczema and the prevalence of specified allergies in pre-defined periods, logistic regression analyses were performed and odds ratios (OR) for period prevalence are reported. Cumulative incidence was estimated by the life table method [17] and analyzed by generalized estimating equations (GEE) [18] using a complementary log-log link and independent correlation structure in PROC GENMOD. The results are presented as relative risks (RR) for the specified contrasts (Full breastfeeding in comparison to randomized formula feeding for the I-cohort and to non-full breastfeeding for the NI-cohort).

To examine the course of allergies, i.e., period prevalences from the 5th to 20th years, considering the status of early eczema and the mode of milk feeding, longitudinal analyses (GEE models with logit link, PROC GENMOD) were performed. The backward selection method (threshold *p* < 0.05) was used to find the best fit for the data and started with all two-factor interaction terms between the terms: time-period, status of early eczema, and mode of milk feeding. Accordingly, the interaction term between early eczema and breastfeeding which would indicate a potential rebound effect was tested for every outcome in every cohort. Only significant interaction terms were retained in the final model. The main factors of interest, i.e., milk feeding and early eczema as well as time period and the set of confounders were fixed in the models (and excluded from elimination process). Results from the final models are given as OR for the interesting terms.

The analyses were conducted for each cohort separately and the models were adjusted for a fixed set of known risk factors or confounders. To avoid multicollinearity the chosen set was reduced to family history of the corresponding outcome (eczema, asthma, and allergic rhinitis, respectively) and heredity of family allergy in the cohorts with family risk of atopy (I-cohort and NI FH+) as well as sex, study region, parental education and older siblings in all cohorts. Parental education was used as a proxy for socio-economic status and study region as a proxy for environmental determinants. Results of the adjusted models are given as adjusted OR or RR (aOR, aRR). *p* values less than 0.05 were considered statistically significant and estimates of OR and RR were given with 95% confidence intervals (95%CI).

## 3. Results

### 3.1. Study Population and Characteristics

For the present analysis, all participants with complete information on early eczema were considered (Table S1). Of the 2252 recruited infants in the I-cohort the status of eczema during the first three years was available for 1661. The NI-cohort comprises 2397 infants, 40 of whom were excluded from the analysis on effects of milk feeding due to missing information. Further details regarding number of subjects at the different stages of follow-ups are presented in Table S1.

Early eczema was more prevalent in children “at risk”: While the prevalence was similar in children with positive family history (FH+) in the I- and NI-cohorts (I-cohort: 453 children (27.3%), NI-cohort: 207 (25.7%)), children in the NI-cohort without family history less often develop early eczema (FH−, 240 children (15.1%), chi-square test *p* < 0.0001 for group differences) (Table 1 and Table S1). Nearly half of the analysis population was fully breastfed for four months, with slightly lower rates in the I-cohort (I-cohort: 44.1%, NI FH+: 51.0%, NI FH−: 47.9%, chi-square test *p* = 0.004 for group differences). Additionally, children in the I-cohort had siblings less often (chi-square *p* < 0.0001 for group differences) while parental education was higher (chi-square *p* < 0.0001 for group differences), the latter especially upon comparison with the NI cohort with negative family history. Additionally, number of siblings and parental education differed in three cohorts (chi-square tests *p* < 0.0001).

### 3.2. Development of Atopic Diseases up to Young Adulthood Depending on Early Eczema during the First Three Years of Life

**Figure 1:**
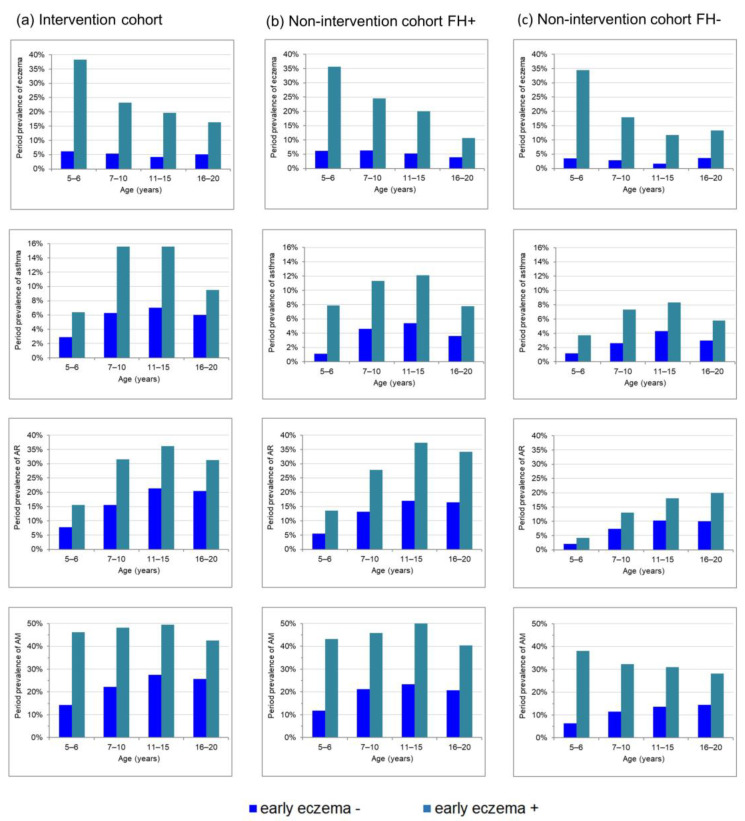
Prevalence of allergic diseases from childhood to young adulthood stratified by eczema during the first 3 years for (**a**) the intervention cohort, (**b**) the non-intervention cohort with family risk of atopy and (**c**) the non-intervention cohort without family risk of atopy.

The illustration of the period prevalence of atopic diseases stratified by the state of early eczema (Figure 1) gives a first description of the potential meaning of early eczema for the development of atopic diseases in later life. Early eczema was apparently accompanied by a higher eczema period prevalence up to young adulthood. Moreover, for asthma and AR, the prevalence was constantly higher in participants with early eczema compared with those without early eczema. This finding was not only observed in subjects with a high risk of atopic diseases, i.e., the I cohort (Figure 1a) and the NI cohort with positive family history (Figure 1b), but also in the NI cohort with a negative family history (Figure 1c).

Statistical analyses using logistic regression models confirmed this conclusion since early eczema was not only a significant predictor of eczema prevalence up to young adulthood, but also of asthma and AR (Table 2). While the highest aORs were generally found for eczema compared with the other atopic phenotypes, the association of early eczema for this phenotype attenuated with increasing age as illustrated by decreasing aORs (Table 2). This is also reflected by decreasing prevalence differences in Figure 1 due to strictly monotonous falling eczema period prevalence with increasing age in those participants with early eczema, but constant levels in those without early eczema (Figure 1). For asthma and AR, a more parallel course was observed in participants with or without early eczema (Figure 1) and also aORs remained relatively constant (Table 2). Longitudinal GEE models with eczema as outcome confirmed that aORs for early eczema decrease with age in the I-cohort and the NI-cohort without family history, while the effect for asthma and also for AR did not change over time in all cohorts (Table 3).

### 3.3. Short- and Long-Term Risk of Allergies in Fully-Breastfeed Children

The 20-year data confirm that the risk reducing effect of breastfeeding on eczema compared with CMF in the I-cohort diminished after early protection and became non-significant when cumulative incidence up to 20 years of life was considered (Table S2). In contrast, high-risk children in the I-cohort fed with eHF-C constantly had an even lower risk of eczema compared with breastfed children not only until 3 years of age, but up to young adulthood. This association remained significant upon adjustment for several confounders. In both groups of the NI-cohort, i.e., children with or without family history, full breastfeeding was not associated with lower cumulative incidences of any atopic outcome compared with non-fully-breastfeeding. Moreover, longitudinal models using period prevalence from five to 20 years as outcome did not reveal general breastfeeding effects on any outcome (Table 3). The adjusted ORs for eczema ranged from 0.92 to 1.1, for asthma from 0.78 to 1.2 and for AR from 0.77 to 1.7 (all n.s.).

### 3.4. Examination of a Potential Rebound Effect in Fully-Breastfed Children without Early Eczema

**Figure 2:**
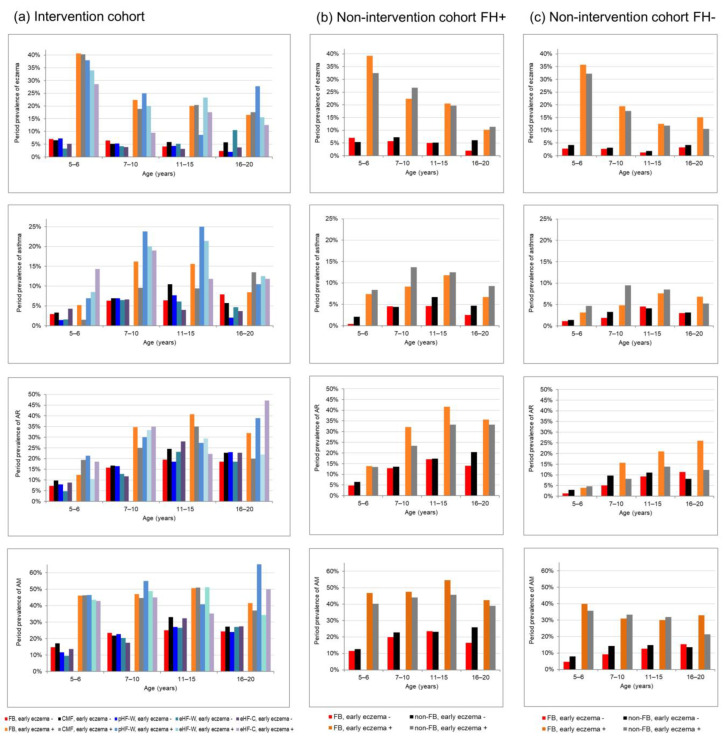
Prevalence of allergic diseases from childhood to young adulthood by eczema during the first three years and feeding groups (**a**) in the intervention-cohort stratified for the full breastfeeding group (FB) and for the four groups supplemented only with their randomized formula, (**b**) in the non-intervention-cohort with family risk (FH+) and (**c**) without risk (FH−) both cohorts stratified by full breastfeeding.

If there was a rebound effect of full breastfeeding, a higher period prevalence in later life would be expected in fully breastfed children without early eczema compared to their counterparts who were fed at least partly with CMF. In general, descriptive illustrations of crude period prevalence in Figure 2 do not indicate such higher period prevalence in fully breastfed children without early eczema (red bars) compared with CMF fed children without early eczema (black bars). Eczema prevalence was slightly higher in the period between 5 and 10 years in the I-cohort (Figure 2a, red bar and black bar) and up to 6 years of age in the NI-cohort in children with family history (Figure 2b) only.

Statistical analyses using longitudinal models included an interaction term between infant milk feeding and early eczema in order to examine the hypothesis of early eczema as a potential modifier (Table 3). These models confirm the impression from the descriptive evaluation of crude prevalence in Figure 2: The analyses did not reveal a significant modifying effect of early eczema on milk feeding effects on the development of eczema, asthma and AR in children with family history of allergies. Differences in breastfeeding effects depending on early eczema were only observed for AR in children without family risk of allergic diseases (NI cohort FH−, *p* for interaction = 0.04): in those without early eczema breastfeeding tended to be associated with lower long-term AR risk compared with CMF (OR= 0.77, 95%CI (0.53–1.1)), while breastfeeding in those with early eczema was associated with a (non-significantly) higher rhinitis risk compared with CMF (OR = 1.7, 95%CI (0.84–3.4), Table 3). Sensitivity analyses including introduction in solid foods as additional covariate did not substantially change these results (results not shown).

**Table 3:** Association of full breastfeeding with allergic diseases from 5 to 20 years under consideration of status of eczema during first three years. Results from adjusted longitudinal GEE models ^#^ for the intervention cohort and non-intervention cohort with family risk of atopy (FH+) and without family risk of atopy (FH−).

		Intervention Cohort (*N* = 1661)	Non-Intervention FH+ (*N* = 792)	Non-Intervention FH− (*N* = 1565)
Eczema				
Interaction feeding with early eczema *	*p*-value	0.987	0.738	0.174
Final Model		M1	M2	M1
FB vs. CMF/non FB	aOR (95%CI)	1.1 (0.72–1.6)	0.92 (0.62–1.3)	0.94 (0.65–1.3)
eHF-C vs. CMF	aOR (95%CI)	0.64 (0.34–1.2)	−^1^	−^1^
Interaction early eczema with time-periods	*p*-value	0.002	–	0.006
Early eczema effect at 5–6th years	aOR (95%CI)	8.8 (6.3–12.3)		14.4 (9.2–22.7)
Early eczema effect at 7–10th years	aOR (95%CI)	4.7 (3.2–7.0)		7.7 (4.4–13.4)
Early eczema effect at 11–15th years	aOR (95%CI)	4.9 (3.1–7.7)		8.6 (4.2–17.4)
Early eczema effect at 16–20th years	aOR (95%CI)	3.2 (2.0–5.1)		3.9 (2.1–7.4)
Early eczema effect	aOR (95%CI)	-	5.0 (3.4–7.4)	-
Time-period 7–10th vs. 5–6th years	aOR (95%CI)		0.76 (0.57–1.02)	
11–15th vs. 5–6th years	aOR (95%CI)		0.58 (0.42–0.80)	
16–20th vs. 5–6th years	aOR (95%CI)		0.34 (0.21–0.52)	
Asthma				
Interaction feeding with early eczema *	*p*-value	0.405	0.698	0.923
Final model		M2	M2	M2
FB vs. CMF/non FB	aOR (95%CI)	1.2 (0.72–2.1)	0.78 (0.45–1.3)	0.95 (0.59–1.5)
eHF-C vs. CMF	aOR (95%CI)	1.1 (0.52–2.1)	−^1^	−^1^
Early eczema effect	aOR (95%CI	2.2 (1.6–3.1)	2.7 (1.6–4.9)	2.4 (1.4–4.1)
Time-period 7–10th vs. 5–6th years	aOR (95%CI)	2.5 (1.9–3.3)	2.4 (1.6–3.6)	2.0 (1.3–3.1)
11–15th vs. 5–6th years	aOR (95%CI)	2.7 (2.0–3.6)	2.8 (1.7–4.5)	3.1 (2.0–4.9)
16–20th vs. 5–6th years	aOR (95%CI)	1.9 (1.4–2.7)	1.7 (1.0–3.0)	2.2 (1.3–3.9)
AR				
Interaction feeding with early eczema *	*p*-value	0.471	0.186	0.040
Final model		M2	M2	M3
FB vs. CMF/non FB	aOR (95%CI)	0.96 (0.68–1.3)	0.98 (0.71–1.4)	-
eHF-C vs. CMF	aOR (95%CI)	1.1 (0.69–1.7)	−^1^	−^1^
FB vs. non FB at early eczema -	aOR (95%CI)			0.77 (0.53–1.1)
FB vs. non FB at early eczema +	aOR (95%CI)			1.7 (0.84–3.4)
Early eczema effect	aOR (95%CI	2.1 (1.7–2.7)	2.7 (1.9–3.8)	
Early eczema effect at FB	aOR (95%CI)			2.7 (1.7–4.5)
Early eczema effect at non FB	aOR (95%CI)			1.2 (0.71–2.2)
Interaction feeding with time-periods	*p*-value	-	-	0.004
FB vs. non FB at 5 –6th years	aOR (95%CI)			0.68 (0.33 –1.5)
FB vs. non FB at 7–10th years	aOR (95%CI)			0.90 (0.55–1.5)
FB vs. non FB at 11–15th years	aOR (95%CI)			1.3 (0.81–2.0)
FB vs. non FB at 16–20th years	aOR (95%CI)			2.2 (1.3–3.6)
Time-period 7–10th vs. 5–6th years	aOR (95%CI)	2.3 (1.9–2.8)	2.5 (1.9–3.4)	
11–15th vs. 5–6th years	aOR (95%CI)	3.3 (2.7–4.0)	3.6 (2.7–4.9)	
16–20th vs. 5–6th years	aOR (95%CI)	2.9 (2.4–3.6)	3.4 (2.4–4.8)	
AM				
Interaction * feeding with early eczema	*p*-value	0.558	0.169	0.177
Final Model		M1	M1	M4
FB vs. CMF/non FB	aOR (95%CI)	0.96 (0.72–1.3)	1.0 (0.76–1.4)	−
eHF-C vs. CMF	aOR (95%CI)	0.90 (0.61–1.3)	−^1^	−^1^
Interaction feeding with time-periods	*p*-value	−	−	0.040
FB vs. non FB at 5–6th years	aOR (95%CI)			0.81 (0.55−1.2)
FB vs. non FB at 7–10th years	aOR (95%CI)			0.70 (0.50–0.99)
FB vs. non FB at 11–15th years	aOR (95%CI)			0.90 (0.64–1.3)
FB vs. non FB at 16–20th years	aOR (95%CI)			1.3 (0.92–1.9)
Interaction early eczema with time-periods	*p*-value	0.001	0.027	0.001
Early eczema effect at 5–6th years	aOR (95%CI)	5.2 (3.9–6.9)	5.5 (3.6–8.4)	9.1 (6.2–13.4)
Early eczema effect at 7–10th years	aOR (95%CI)	3.2 (2.4–4.2)	3.0 (2.0–4.5)	3.6 (2.4–5.4)
Early eczema effect at 11–15th years	aOR (95%CI)	2.5 (1.9–3.3)	3.2 (2.2–4.8)	2.8 (1.9–4.2)
Early eczema effect at 16–20th years	aOR (95%CI)	2.1 (1.5–2.8)	2.6 (1.6–4.2)	2.2 (1.4–3.4)

^1^ not to be determined in the non-intervention cohort; ^#^ All models were adjusted for family history of corresponding disease, heredity of family allergy, sex, study region, siblings, parental education in the intervention and non-intervention FH+ cohort and for sex, study region, siblings, parental education in the non-intervention FH− cohort; * test of interaction-term whether early eczema modify the feeding association, started with all two-factor interaction terms and used backward selection modelling. *p*-values for the interaction between feeding and early eczema were derived from the last step, where the interesting term was deleted or from final model. Final models: Only significant interaction terms were retained as a result from backward selection modelling. The main interesting factors milk feeding and early eczema as well as time-period and the set of confounders were fixed in the models (and excluded from elimination process). M1: model includes variables for time-periods, status of early eczema, feeding groups and the interaction of status of early eczema with time-periods; M2: model includes variables for time-periods, status of early eczema, feeding groups; M3: model includes variables for time-periods, status of early eczema, FB feeding, interaction of status of early eczema with feeding and interaction of feeding with time-periods; M4: Model includes variables for time-periods, status of early eczema, feeding groups and the interaction of feeding with time-periods and interaction of status of early eczema with time-periods.

## 4. Discussion

Using data from the GINIplus study up to 20 years of age, we examined whether early eczema is a determinant of the course of allergic disease until early adulthood. Additionally, we examined the hypothesis of a potential rebound effect in breastfed children after initial protection against allergies in young childhood. Our analysis showed that early eczema is linked with a higher risk of all types of allergic diseases over the course of childhood up to 20 years of age. Odds ratios were generally highest for eczema, but decrease with increasing age. Additionally, our analysis provides evidence that preventive effects of full breastfeeding on eczema are restricted to subgroups and early childhood, attenuate over time and do not persist until young adulthood. However, since early eczema was not observed to modify the association between milk feeding and allergy risk, we did not find evidence of a rebound effect after initial protection in young childhood.

Our present analysis shows that early eczema is not only associated with eczema in later life, but also with rhinitis and asthma. This finding could be related to a “multimorbid” allergic cluster, which was identified as one of seven different clusters of allergic disease development from birth until adolescence in a combined analysis of the German GINIplus and LISAplus studies [19]. Our data also showed that eczema prevalence decreases over time in those subjects with early eczema, indicating a natural remission, i.e., an “early−resolving dermatitis” cluster [19]. Interestingly, Kilanowski et al. identified sex, parental history of allergies, and pet exposure as early life determinants for the different clusters, while breastfeeding did not differ between allergic cases and controls in their analysis [19].

Our finding that full breastfeeding does not decrease the risk of allergic diseases across subgroups and at different ages is completely in line with conclusions stated in the current S3-guidelines Allergy Prevention in Germany [2]. Focusing on data from a particularly vulnerable group, i.e., children with a family risk for allergic diseases, the GINIplus study indicates that full breastfeeding might only have transient preventive effects on allergy in early life. Indeed, the initial reduction in cumulative incidence of eczema lost its significance when considering data up to young adulthood, while lower risks of rhinitis or asthma were neither observed in the short- nor in the long-term in this subgroup. The Global Initiative for Asthma (GINA) recently also concluded that breastfeeding decreases wheezing episodes in early life, but may not prevent the development of persistent asthma [20]. Interestingly, the hypothesis of attenuating effects on eczema and/or asthma does not seem to be transferable to infant nutrition in general. Using data from the I-cohort, we recently examined effects of different types of formulae [12]. We observed that the cumulative incidence of eczema until young adulthood was reduced in children fed with eHF-C or pHF-W compared to the CMF group. Additionally, asthma prevalence between 16 and 20 years was significantly lower in both groups compared to CMF.

Two very recent studies using data from large-scale population-based studies, i.e., National Health and Nutrition Examination Survey (NHANES, 833 cases and 5167 controls) [21] and UK Biobank (7157 cases with childhood-onset asthma and 158,253 controls) [22], could not yet be considered in the above-mentioned guidelines. Both showed potential protective effects: Breastfed children from the UK Biobank less frequently developed asthma until 12 years of age compared to their counterparts who were not breastfed [22]. The second study showed that in three to six year-old children participating in NHANES who were exclusively breastfed for at least four to six months asthma risk was reduced by 31% compared with children never breastfed [21]. Interestingly, Chen et al. also reported that protective effects seem to diminish in older children [21].

We hypothesized that diminishing protective effects could represent a rebound effect in fully breastfed children. This could be explained through less exposure to potential allergens in cow’s milk formula which in turn might influence the development of tolerance and therefore affect development of allergic diseases in the long-term. Another explanation for the reduced protective effect could be that the early exposure to immune components found in breast milk such as antibodies, growth factors, cytokines, antimicrobial compounds, and specific immune cells [23] might only protect against allergies until young childhood. Over the years, other factors including environmental factors such as air pollution are likely to have an increasing effect on the immune system, overriding nutritional effects during infancy. However, longitudinal analyses over the course of childhood in our study did not generally support the hypothesis of a rebound effect. To the best of our knowledge, a rebound effect in fully breastfed children regarding allergies has not yet been examined in other studies. Most studies are limited in that they focused on relatively early periods of life potentially hampering identification of a rebound effect. Up to now, the 16-years follow-up of the cluster-randomized controlled study PROBIT conducted in Belarus provides the longest-term insights into this topic [24]. Focusing on effects of breastfeeding promotion on health development, PROBIT also assessed several allergy outcomes at the age of 16 years with mixed results: Flohr et al. reported a 50% risk reduction regarding flexural eczema on skin examination at 16 years of age, but no significant associations with self-reported eczema or asthma symptoms in the past year [24]. Rebound effects of breastfeeding were not reported.

While the findings in PROBIT based on self-reports are comparable with our GINIplus data, the reduction in the prevalence of flexural eczema based on clinical examination even seems to indicate long-term protective effects of full breastfeeding [24]. Accordingly, studies using self-reports on eczema such as GINIplus might underestimate long-term effects of breastfeeding. However, partly conflicting results between GINIplus and PROBIT may also be explained by differences in study design and populations. PROBIT was conducted in a setting with a low risk of allergic diseases: Prevalence of family risk of allergic diseases (5.2% in the intervention group and 3.7% in the control group) as well as the prevalence of eczema in study participants themselves (<1% in both study groups upon skin examination as well as self-reported symptoms) [24] were substantially lower compared with the population in our GINIplus study. It might be speculated that breastfeeding is more relevant in the long-term in populations where genetic risk and/or exposure to other risk factors is less pronounced. Even if protective effects of breastfeeding on eczema are restricted to early childhood in populations such as GINIplus, these effects could still be relevant for long-term health beyond allergies. A previous analysis of the GINI study revealed a higher risk for mental health problems at the age of 10 years in children with infant-onset eczema even if eczema was limited to infancy compared with children never diagnosed as having eczema [25]. Accordingly, full breastfeeding could have indirect long-term effects on mental health by protecting against infant onset eczema.

While our study in general did not reveal long-term effects of breastfeeding on allergies, an interesting finding is that we also observed potential long-term benefits in a subgroup with low allergy risk: In children without family risk of allergies and without early eczema, a subgroup that has not been in the focus so far, full breastfeeding was associated with a lower period prevalence of rhinitis between five and 20 years of age. Considering that this finding missed significance, more studies are needed to confirm a potential long-term preventive effect of breastfeeding particularly in children with a negative family history of atopy.

A strength of our analysis is that we considered data not only until adolescence, but up to young adulthood. Compared with previous results from GINIplus considering data until adolescence only [8], slightly decreasing effect sizes as well as the lack of significance regarding cumulative incidence of eczema in the current analysis indicate that the relevance of breastfeeding might attenuate further in young adulthood. A further strength of GINIplus is that the repeated allergy assessment is based on the same set of questions throughout the ages. Accordingly, these data provide the opportunity to conduct longitudinal data analysis which not only increases the statistical power, but also enables the evaluation of rebound effects of breastfeeding over time.

Several limitations of the study need to be considered. First of all, although GINIplus included an interventional study cohort, this part of the study was not a priori designed to assess the effects of breastfeeding. In the interventional study, mothers were encouraged to exclusively breast-feed for at least 4 months and preferably 6 months. The decision to include the specific study formula in their children’s diet was exclusively made by the parents. In the NI cohort, parents did not receive dietary recommendations. Accordingly, the present data analysis represents an observational study design, which hampers conclusions on causality due to the general risk of reverse causation and residual confounding. Regarding the relationship between breastfeeding and atopic diseases, Lowe et al. hypothesized that the protective effects of breastfeeding could be masked due to prolonged breastfeeding in children with early signs of atopic diseases [26]. Accordingly, our study may underestimate the true preventive effects of breastfeeding. Regarding the risk of residual confounding, we observed that early eczema was associated with both, exposition (i.e., full breastfeeding) and outcome (i.e., allergic diseases). Accordingly, future studies investigating allergy prevention effects of breastfeeding should consider early eczema as potential mediator or confounder. Last but not least, the GINIplus sample is not representative for children in Germany, which might restrict the external validity of our results. However, e.g., full-breastfeeding prevalence was comparable to the representative SuSe I study conducted in 1997: in SuSe I, a prevalence of 48.5 % of breastfeeding only (exclusive and predominant breastfeeding, i.e., breastfeeding plus liquids) was found in West Germany at the age of 4 months [27]. We therefore believe that our results give a good impression on the relevance of breastfeeding for allergy prevention in the general population.

## 5. Conclusions

Preventive effects of breastfeeding seem to attenuate with increasing age at least in children with family risk of allergies. However, there is no rebound effect in fully breastfed children without early eczema with regard to allergies in later life. Accordingly, data from GINIplus confirm that breastfeeding should be recommended in terms of allergy prevention—at least for beneficial effects in early life. Potential long-term protection against rhinitis in children with low family risk of allergies needs to be confirmed in future studies.

## Figures and Tables

**Table 1:** Characteristics of the analysis population, stratified by study cohorts * and eczema during the first three years.

		Intervention Cohort			Non-Intervention Cohort FH+			Non-Intervention Cohort FH−		
		Early Eczema −	Early Eczema +		Early Eczema −	Early Eczema +		Early Eczema −	Early Eczema +	
		*N* = 1208	*N* = 453	Chi^2^ test	*N* = 597	*N* = 207	Chi^2^ test	*N* = 1353	*N* = 240	Chi^2^ test
		*n* (%)	*n* (%)	*p*-value	*n* (%)	*n* (%)	*p*-value	*n* (%)	*n* (%)	*p*-value
Family history of allergy	no	0	0		0	0		1353 (100)	240 (100)	-
	single	846 (70.0)	291 (64.2)	0.024	503 (84.3)	159 (76.8)	0.016			
	double	362 (30.0)	162 (35.8)		94 (15.7)	48 (23.2)				
Family risk for	eczema	419 (34.7)	249 (55.0)	<0.001	155 (26.0)	96 (46.4)	<0.001	-	-	
	asthma	329 (27.2)	141 (31.1)	0.117	108 (18.1)	40 (19.3)	0.693	-	-	
	AR	1033 (85.5)	370 (81.7)	0.055	453 (75.9)	154 (74.4)	0.669	-	-	
Sex	male	615 (50.9)	244 (53.9)	0.284	294 (49.2)	114 (55.1)	0.149	687 (50.8)	119 (49.6)	0.733
Study region	Munich	635 (52.6)	239 (52.8)	0.944	364 (61.0)	122 (58.9)	0.606	469 (34.7)	104 (43.3)	0.010
Siblings ^#^	0	719 (59.5)	252 (55.6)	0.246	262 (43.9)	78 (37.7)	0.256	675 (49.9)	141 (58.8)	0.037
	1	373 (30.9)	145 (32.0)		241 (40.4)	96 (46.4)		520 (38.4)	78 (32.5)	
	>1	113 (9.4)	53 (11.7))		94 (15.7)	33 (15.9)		158 (11.7)	21 (8.8)	
Parental education ^#^	low (<10 years)	76 (6.3)	28 (6.2)	0.707	59 (9.9)	19 (9.2)	0.923	176 (13.0)	34 (14.2)	0.641
	middle (10–12 years)	328 (27.2)	132 (29.1)		166 (27.8)	60 (29.0)		462 (34.1)	74 (30.8)	
	high (>12 years)	804 (66.6)	292 (64.5)		372 (62.3)	128 (61.8)		714 (52.8)	129 (53.8)	
Full breastfeeding for 4 months ^#^		533 (44.1)	200 (44.2)	0.992	302 (51.5)	102 (49.5)	0.618	633 (47.5)	116 (50.0)	0.479

FH+: children with family risk of atopy; FH−: children without family risk of atopy; early eczema+: children with eczema up to the 3rd year of life; early eczema−: children without eczema up to the 3rd year of life; * chi^2^ test revealed significant differences between the three cohorts for all parameters (all *p* < 0.01) except for sex (*p* = 0.797). ^#^ 3 missing values regarding siblings, 5 missing values regarding parental education; 12 and 28 missing values regarding full breastfeeding in the Non-intervention FH+ and Non-intervention FH−.

**Table 2:** Association between eczema during the first three years and prevalence of allergies in life periods up to age 20 years in the intervention-cohort and for the non-intervention-cohort with family risk of atopy family risk of atopy (FH+) and without family risk of atopy (FH−). OR ^a^ and adjusted OR (aOR ^b,c^) with 95%CI from logistic models for those with eczema during the first three years in comparison to those without.

		**Prevalence eczema**							
		**5 to 6th Year**		**7 to 10th Year**		**11 to 15th Year**		**16 to 20th Year**	
Intervention	OR	9.5	(6.9–13.2)	5.3	(3.6–7.8)	5.5	(3.6–6.6)	3.6	(2.3–5.8)
	aOR ^b^	8.7	(6.2–12.2)	5.0	(3.4–7.6)	5.0	(3.2–7.9)	3.4	(2.1–5.5)
Non-intervention FH+	OR	8.5	(5.2–13.7)	4.8	(2.8–8.2)	4.6	(2.6–8.1)	2.9	(1.3–6.5)
	aOR ^b^	8.1	(4.9–13.4)	4.4	(2.5–7.6)	4.1	(2.2–7.4)	2.4	(1.0–5.6)
Non-intervention FH−	OR	14.6	(9.4–22.6)	7.4	(4.3–12.8)	8.3	(4.1–16.9)	4.0	(2.2–7.5)
	aOR ^c^	14.7	(9.5–22.8)	7.4	(4.2–12.9)	8.2	(4.0–16.9)	4.7	(2.4–9.1)
		**Prevalence asthma**							
		**5 to 6th year**		**7 to 10th year**		**11 to 15th year**		**16 to 20th year**	
Intervention	OR	2.3	(1.3–4.0)	2.7	(1.8–4.1)	2.5	(1.6–3.7)	1.6	(1.0–2.7)
	aOR ^b^	2.1	(1.2–3.8)	2.6	(1.7–4.0)	2.4	(1.6–3.6)	1.6	(0.95–2.7)
Non-intervention FH+	OR	7.4	(2.8–19.8)	2.6	(1.4–5.1)	2.4	(1.3–4.5)	2.2	(0.93–5.4)
	aOR ^b^	6.8	(2.5–18.6)	2.5	(1.3–5.0)	2.2	(1.2–4.3)	1.9	(0.78–4.8)
Non-intervention FH−	OR	3.0	(1.2–7.6)	3.0	(1.5–6.1)	2.0	(1.1–3.9)	2.0	(0.87–4.5)
	aOR ^c^	2.9	(1.1–7.3)	3.2	(1.5–6.5)	2.1	(1.1–4.1)	2.1	(0.89–4.7)
		**Prevalence allergic rhinitis/hay fewer**							
		**5 to 6th year**		**7 to 10th year**		**11 to 15th year**		**16 to 20th year**	
Intervention	OR	2.2	(1.5–3.2)	2.5	(1.9–3.4)	2.1	(1.6–2.8)	1.8	(1.3–2.4)
	aOR ^b^	2.2	(1.5–3.1)	2.6	(1.9–3.5)	2.1	(1.6–2.8)	1.7	(1.2–2.4)
Non-intervention FH+	OR	2.7	(1.5–4.8)	2.5	(1.6–4.0)	2.9	(1.9–4.4)	2.6	(1.6–4.3)
	aOR ^b^	2.8	(1.5–5.0)	2.4	(1.6–3.9)	2.9	(1.9–4.4)	2.6	(1.6–4.3)
Non-intervention FH−	OR	2.0	(0.89–4.5)	1.9	(1.1–3.2)	1.9	(1.2–3.1)	2.2	(1.4–3.6)
	aOR ^c^	2.0	(0.87–4.5)	1.9	(1.1–3.2)	2.0	(1.2–3.1)	2.1	(1.3–3.4)
		**Prevalence allergic diseases (Eczema, asthma or rhinitis/hay fewer)**							
		**5 to 6th year**		**7 to 10th year**		**11 to 15th year**		**16 to 20th year**	
Intervention	OR	5.1	(3.9–6.7)	3.3	(2.5–4.3)	2.6	(2.0–3.4)	2.1	(1.6–2.9)
	aOR ^b^	5.0	(3.8–6.6)	3.4	(2.5–4.5)	2.6	(2.0–3.5)	2.1	(1.5–2.8)
Non-intervention FH+	OR	5.7	(3.8–8.5)	3.1	(2.1–4.6)	3.3	(2.2–4.9)	2.6	(1.6–4.1)
	aOR ^b^	5.6	(3.6–8.6)	3.1	(2.1–4.7)	3.2	(2.1–4.9)	2.5	(1.6–4.1)
Non-intervention FH−	OR	9.2	(6.3–13.4)	3.7	(2.5–5.4)	2.8	(1.9–4.1)	2.3	(1.5–3.5)
	aOR ^c^	9.1	(6.2–13.4)	3.7	(2.5–5.5)	2.8	(1.9–4.2)	2.3	(1.5–3.5)

^a^ OR of <1 indicates a decreased risk of disease, that is lower risk in the early eczema group than in the compared feeding group, whereas OR >1 indicates higher risk in early eczema group than in the compared non-early eczema group; ^b^ adjusted for family history of corresponding disease, heredity of family allergy, sex, study region, siblings, parental education; ^c^ no family history per definition, therefore adjusted for sex, study region, siblings, parental education.

## Data Availability

Data available on reasonable request due to restrictions of privacy, provided it is consistent with the consent given by the study participants. In some cases, ethical approval can be obtained for the release. Lastly, a data transfer agreement must be accepted and the request must be approved by the studies’ steering committees. Requests should be addressed to MS (marie.standl@helmholtz-muenchen.de).

## Supplementary Materials

The following supporting information can be downloaded at: https://www.mdpi.com/article/10.3390/nu15122780/s1, Table S1: Number of participants during follow-up stratified by eczema during first 3 years and mode of milk feeding in the first 4 months in the intervention cohort and by early eczema and status of full-breastfeeding (FB) in the first 4 months in the non-intervention cohort with family risk of atopy (FH+) and without family risk of atopy (FH−); Table S2: Cumulative incidence of allergic diseases from birth to 20 years of age for fully breastfed infants in comparison with formula fed infants in the intervention-cohort and in comparison with non-fully breastfed infants with family risk of atopy (FH+) and without family risk of atopy (FH−) in the non-intervention-cohort.
