# Supplementary material for: Full Breastfeeding and Allergic Diseases—Long-Term Protection or Rebound Effects?
Source: Nutrients. 2023 Jun 16;15(12):2780. doi: 10.3390/nu15122780 (PMC10301269; doi:10.3390/nu15122780)
Supplement: Supplementary file 1 [file nutrients-15-02780-s001.zip › nutrients-2408839-supplementary.pdf]

Supplementary information

Table S1. Number of participants during follow-up stratified by eczema during first 3 years [1] and mode of milk feeding in the first 4 months in the intervention cohort and by early eczema and status of full-breastfeeding (FB) in the first 4 months in the non-intervention cohort with family risk of atopy (FH+) and without family risk of atopy (FH-).

			Follow−Up Period at Age [Years]				
			**1–3**	**5–6**	**7–10**	**11–15**	**16–20**
Intervention		Early eczema +	453	385	331	326	274
		Early eczema -	1208	1076	930	878	759
Non−intervention, FH+		Early eczema +	207	170	155	155	115
		Early eczema -	597	546	475	454	361
Non−intervention, FH-		Early eczema +	240	202	167	165	140
		Early eczema -	1353	1199	1006	975	775
Intervention	FB	Early eczema +	200	179	156	156	144
		Early eczema -	533	491	440	402	358
	CMF [2]	Early eczema +	78	69	54	54	37
		Early eczema -	135	125	106	105	89
	Phf-W [2]	Early eczema +	42	29	23	25	19
		Early eczema -	159	144	122	121	100
	eHF-W [2]	Early eczema +	56	49	46	45	32
		Early eczema -	146	128	103	102	88
	eHF-C [2]	Early eczema +	30	28	22	20	17
		Early eczema -	138	120	107	100	83
	Non-compliant [4]	Early eczema +	47	31	30	26	25
		Early eczema -	97	68	52	48	41
Non−intervention [3] FH+	FB	Early eczema +	102	83	79	79	60
		Early eczema -	302	280	250	245	205
	Non-FB	Early eczema +	104	86	76	75	54
		Early eczema -	284	255	215	199	150
Non−intervention [3] FH-	FB	Early eczema +	116	103	85	84	75
		Early eczema -	633	571	501	489	402
	Non-FB	Early eczema +	116	92	76	74	59
		Early eczema -	700	610	492	472	361

FB: 4 months fully breastfed, FH+: children with a family history of allergies; FH-: children without a family history of allergies; early eczema+: children with eczema up to the 3rd year of life; early eczema-: children without eczema up to the 3rd year of life; [1] defined by parental reported physician's diagnosis; [2] Per protocol population with known status of early eczema; [3] Non-intervention is reduced by patients where FB feeding is unknown (12 in FH+ and 28 in FH-); [4] either received at least one bottle of a formula which was not allowed according to the study protocol or information on the mode were incomplete.

Table S2. Cumulative incidence of allergic diseases from birth to 20 years of age for fully breastfed infants in comparison with formula fed infants in the intervention-cohort and in comparison with non-fully breastfed infants with family risk of atopy (FH+) and without family risk of atopy (FH-) in the non-intervention-cohort.

			**Eczema 1st to 3rd Year**		**Eczema 1st to 20th Year**		**Asthma 3rd to 20th Year**		**Allergic Rhinitis/ Hay Fever 4th to 20th Year**	
Intervention	CMF	RR	0.72	(0.55–0.94)	0.84	(0.63–1.10)	0.92	(0.61–1.38)	0.91	(0.70–1.20)
		aRR	0.68	(0.52–0.89)	0.77	(0.58–1.02)	1.01	(0.67–1.53)	0.90	(0.68–1.18)
	pHF-W	RR	1.36	(0.97–1.92)	1.32	(0.98–1.78)	0.83	(0.55–1.26)	0.88	(0.67–1.17)
		aRR	1.29	(0.92–1.83)	1.30	(0.96–1.76)	0.97	(0.64–1.48)	0.91	(0.69–1.20)
	eHF-W	RR	0.99	(0.73–1.34)	1.01	(0.76–1.34)	0.98	(0.63–1.52)	1.04	(0.78–1.39)
		aRR	0.98	(0.72–1.34)	1.04	(0.77–1.39)	1.02	(0.64–1.64)	1.06	(0.79–1.42)
	eHF-C	RR	1.67	(1.12–2.47)	1.71	(1.22–2.40)	0.94	(0.60–1.47)	0.96	(0.72–1.28)
		aRR	1.61	(1.08–2.38)	1.67	(1.19–2.36)	1.07	(0.69–1.67)	0.97	(0.73–1.29)
	Non-compliant	RR	0.93	(0.67–1.29)	0.76	(0.55–1.04)	0.99	(0.58–1.70)	0.71	(0.52–0.97)
		aRR	0.91	(0.65–1.28)	0.75	(0.54–1.04)	1.10	(0.63–1.90)	0.71	(0.51–0.98)
Non-intervention FH+	non-FB	RR	0.94	(0.71–1.24)	0.86	(0.66–1.13)	0.65	(0.41–1.05)	0.96	(0.72–1.27)
		aRR	0.88	(0.66–1.17)	0.82	(0.62–1.08)	0.77	(0.48–1.24)	1.00	(0.76–1.33)
Non-intervention FH−	non-FB	RR	1.12	(0.87–1.45)	0.94	(0.75–1.18)	0.74	(0.49–1.10)	0.94	(0.73–1.22)
		aRR	1.14	(0.88–1.48)	0.96	(0.76–1.21)	0.82	(0.55–1.22)	0.96	(0.74–1.24)

RR[#] and adjusted RR (aRR)* with 95%CI from GEE models. [#] RR of < 1 indicates a decreased relative risk of disease, that is lower incidence in the fully breastfed group than in the compared feeding group, whereas RR > 1 indicates higher incidence in fully breastfed group than in the compared formula feeding group; Bold values and bold CI were used for significant adjusted effects. * adjusted for family history of corresponding disease, heredity of family allergy, sex, study region, siblings, parental education; Calculation on incidence was based on 2040 infants in intervention cohort, 902 in Non-intervention FH+ and 1852 in Non-intervention FH-.
